# Supplementary material for: Inferring clonal evolution of tumors from single nucleotide somatic mutations
Source: BMC Bioinformatics. 2014 Feb 1;15:35. doi: 10.1186/1471-2105-15-35 (PMC3922638; doi:10.1186/1471-2105-15-35)

Figure S1: Full partial order plot for SU070

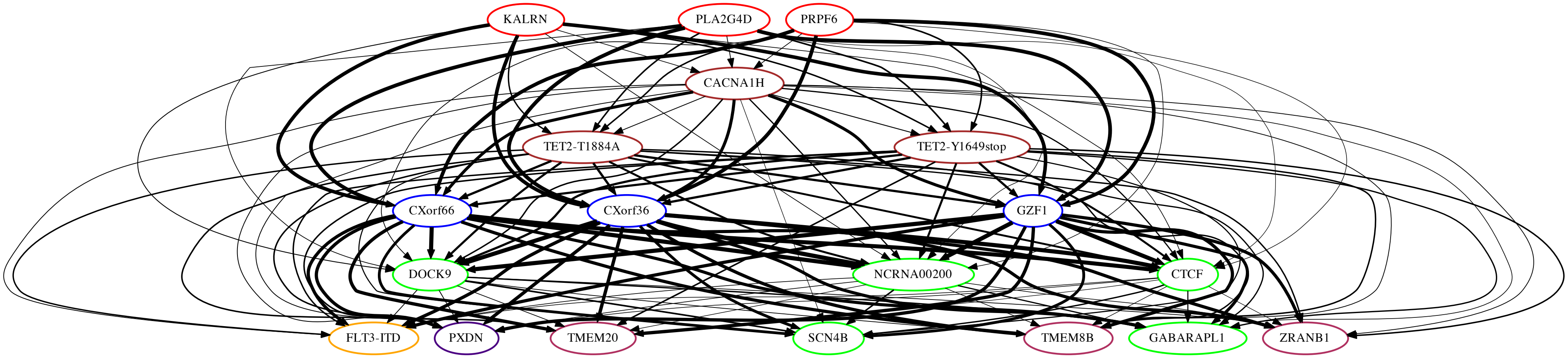

Figure S2: Full partial order plot for SU048

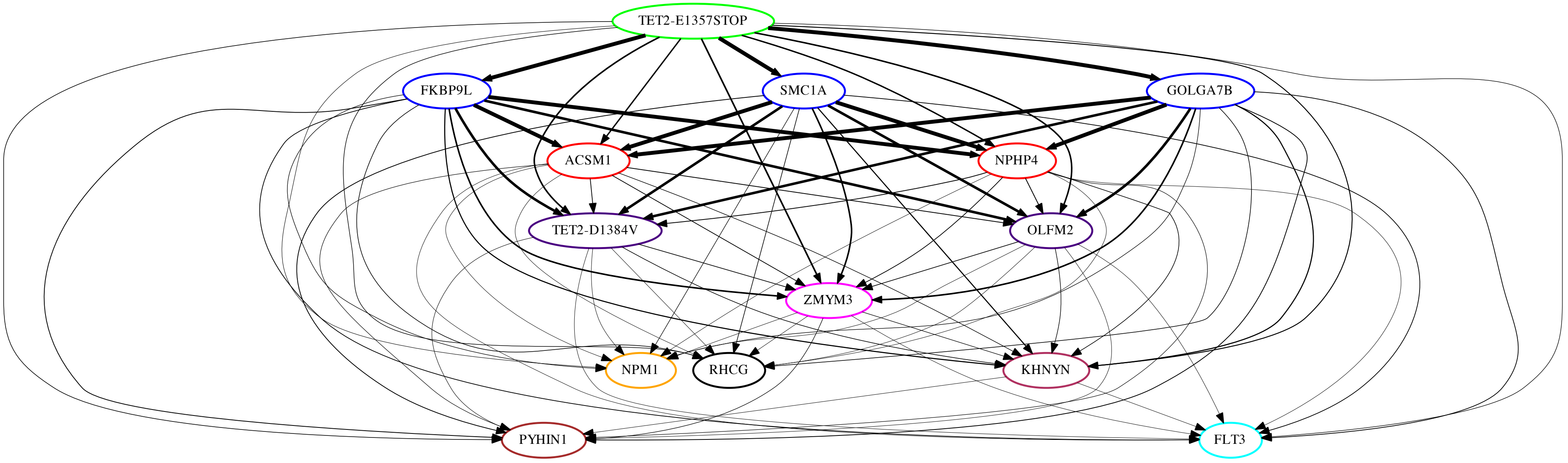

Figure S3: Log likelihood trace and autocorrelation function plots for SU070

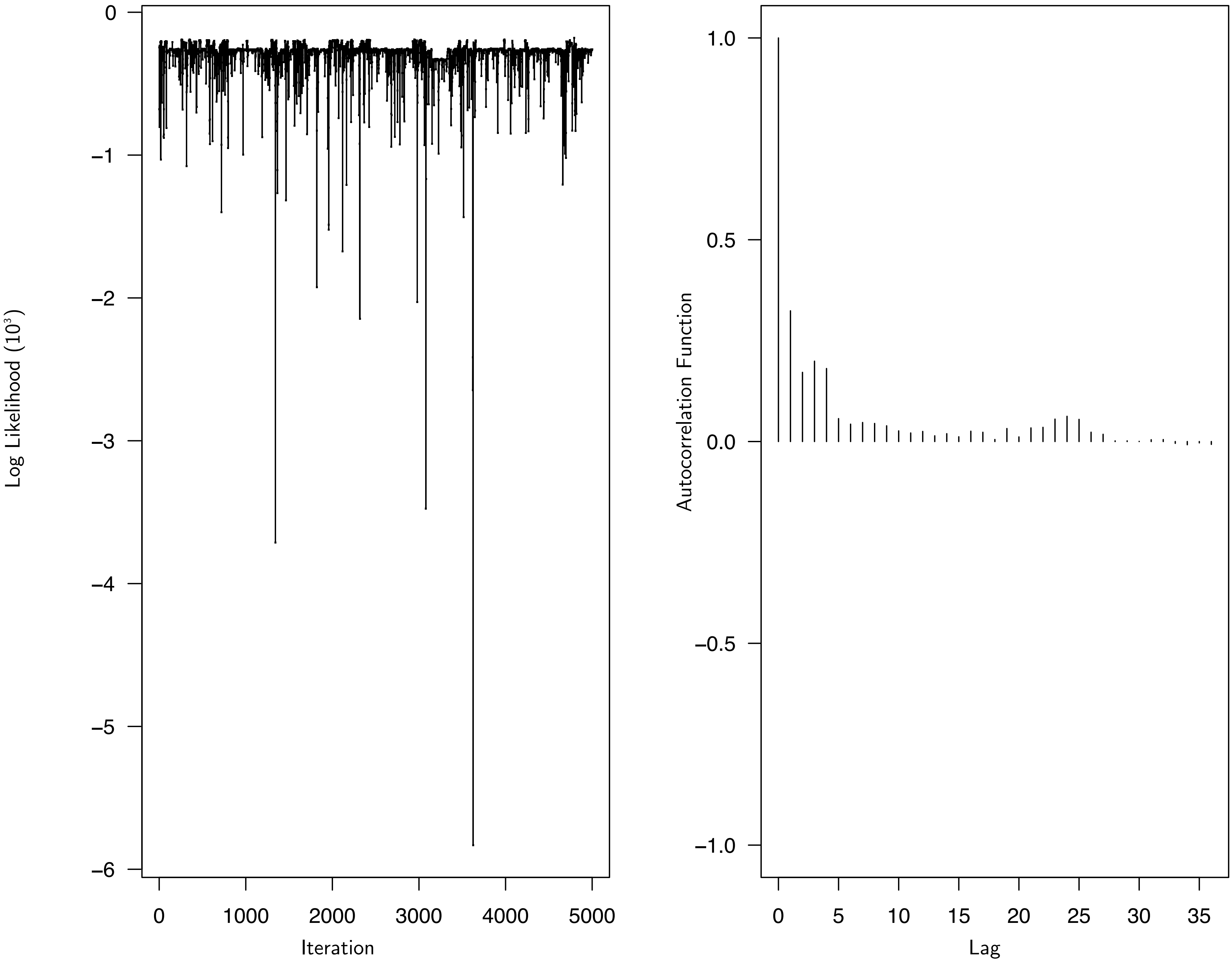

Figure S4: Log likelihood trace and autocorrelation function plots for SU048

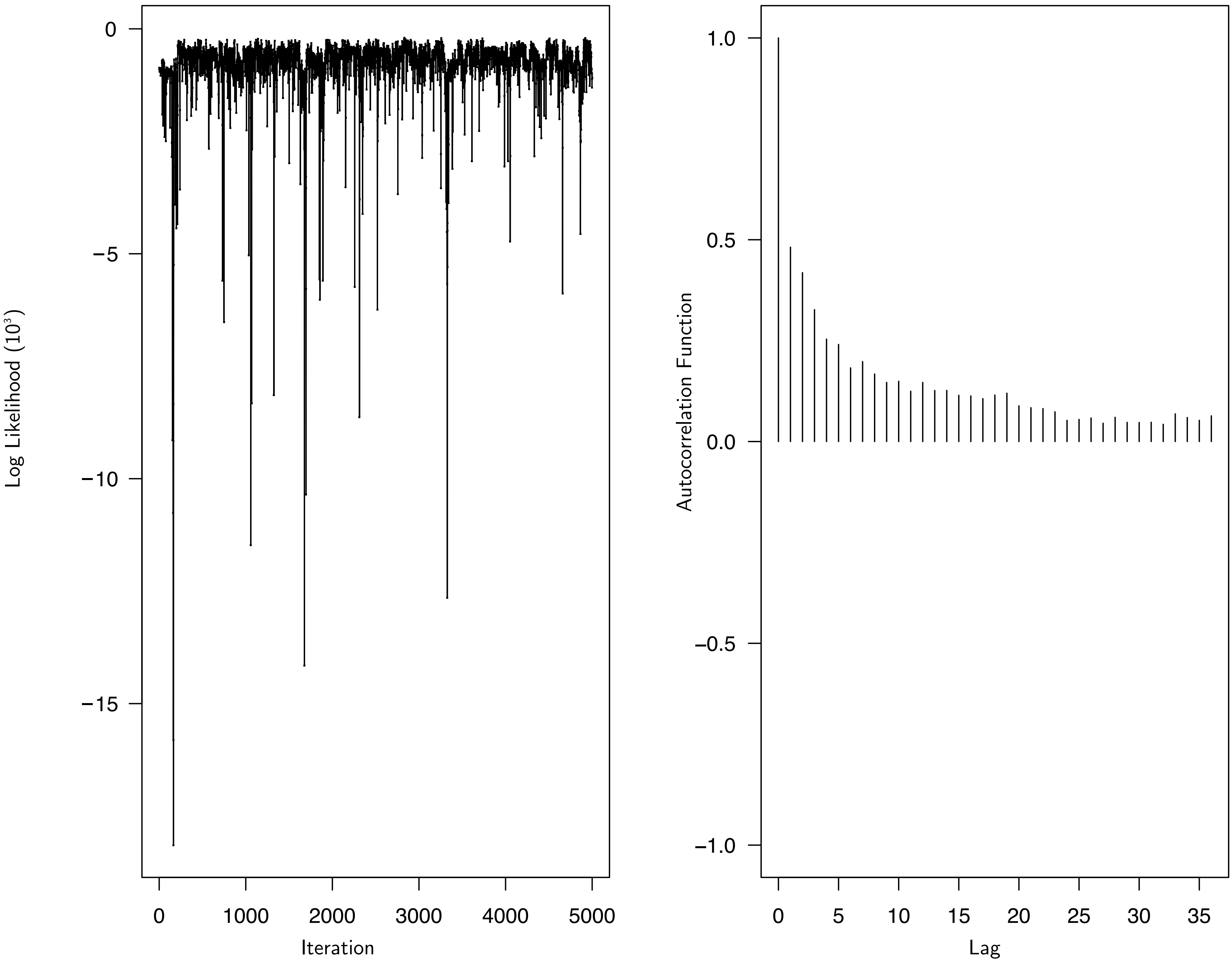

Figure S5: Log likelihood trace and autocorrelation function plots for CLL077

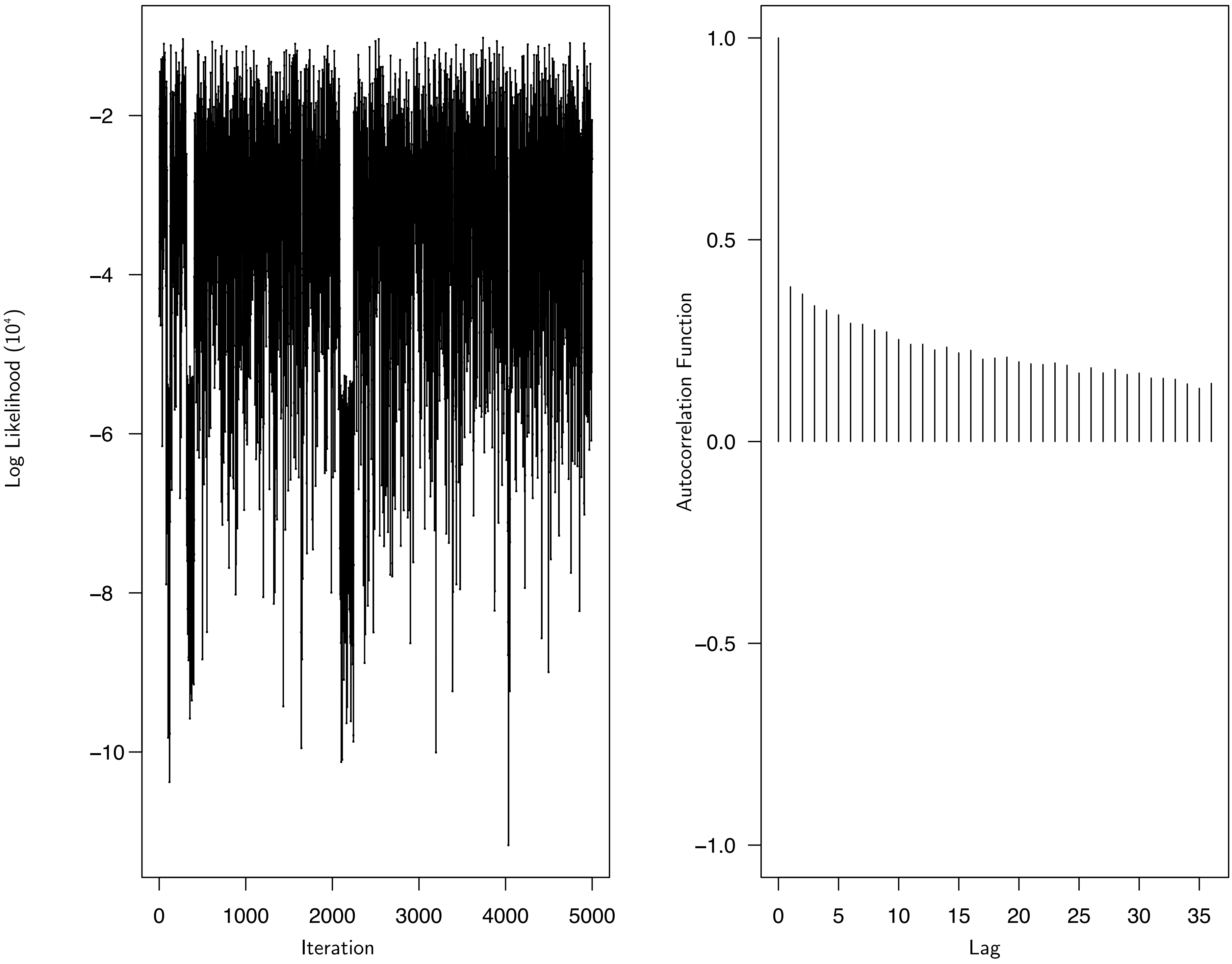

Figure S6: Log likelihood trace and autocorrelation function plots for CLL006

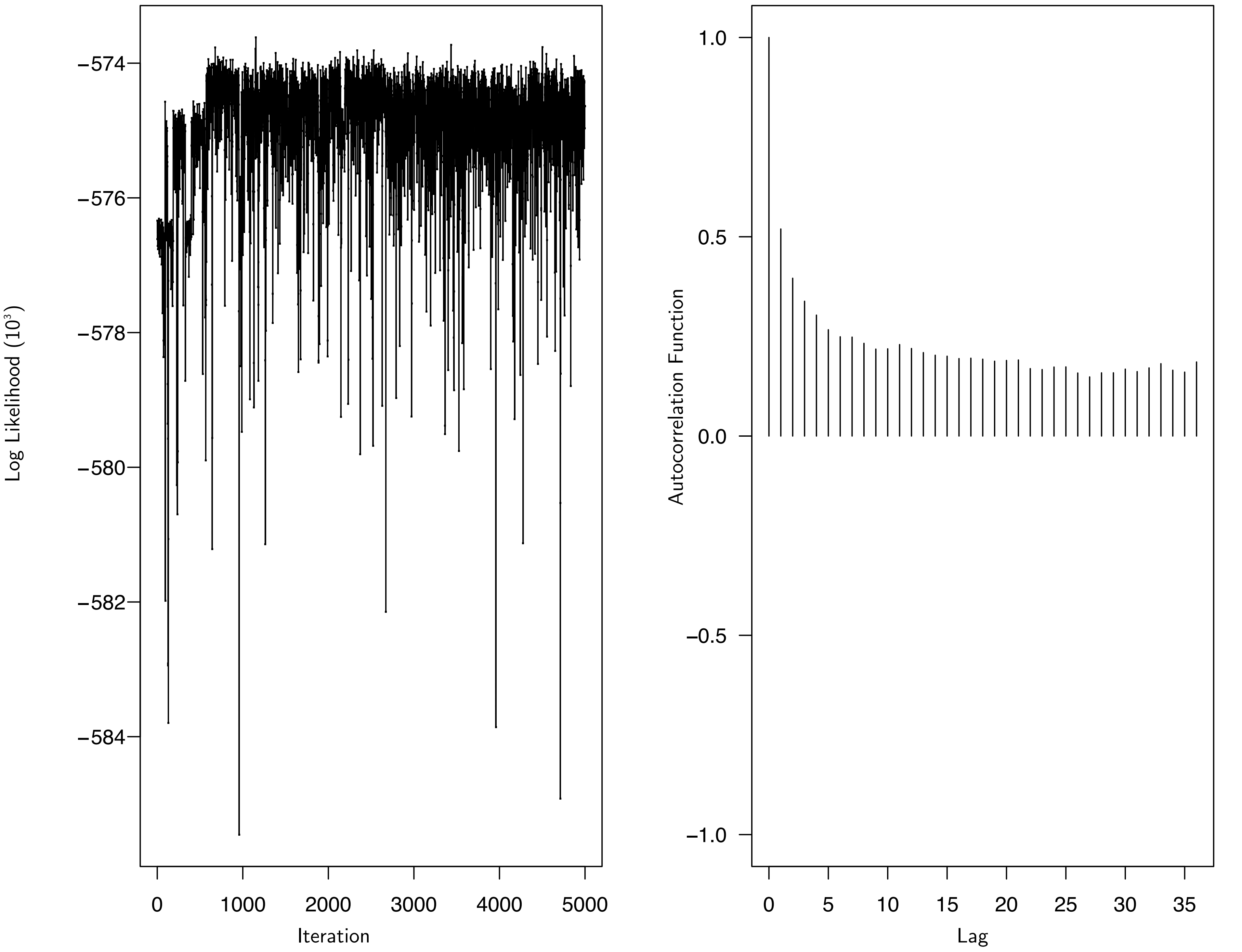

Figure S7: Log likelihood trace and autocorrelation function plots for CLL003

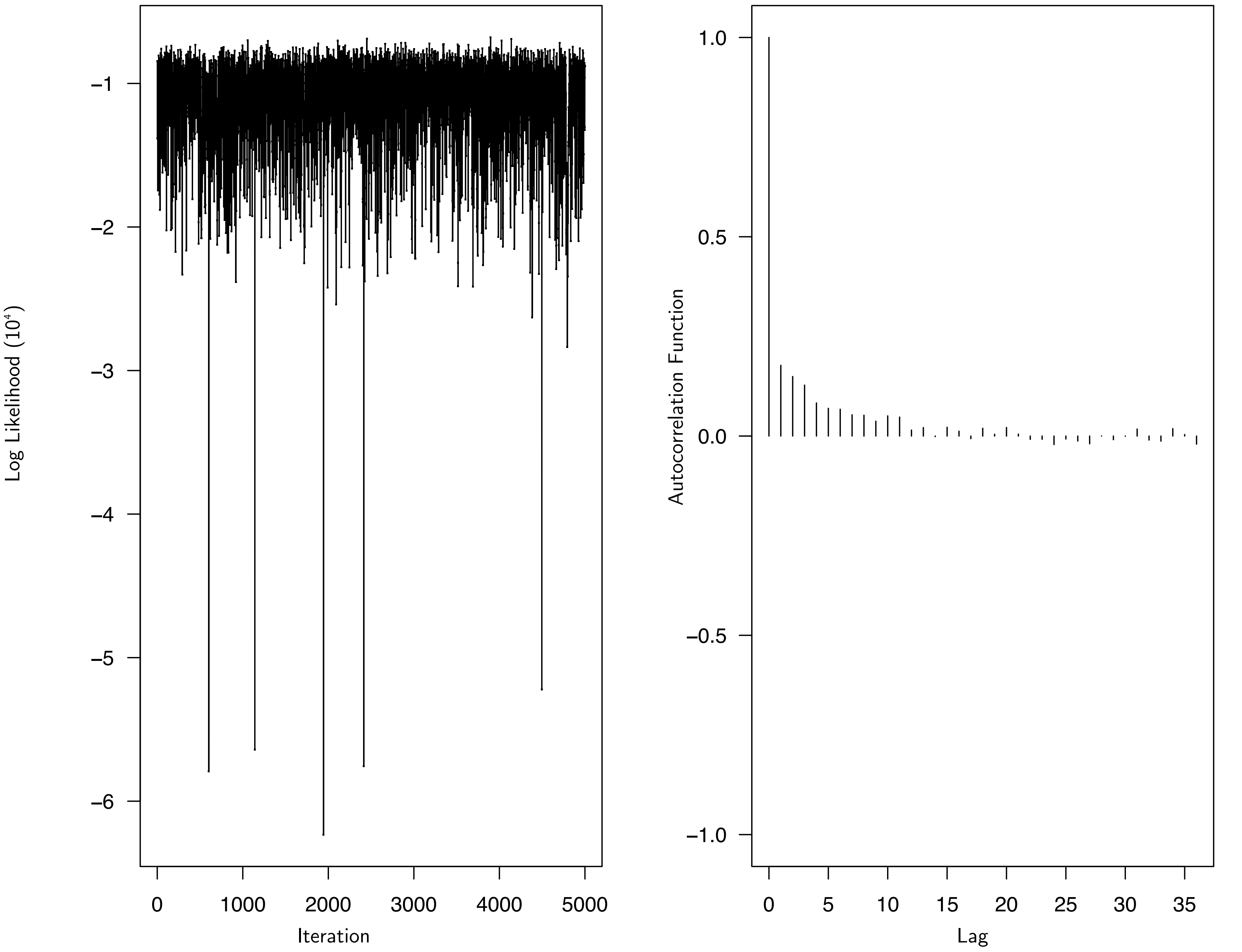

Supplement: Additional file 2 — Supplementary figures. This file contains supplementary Figures S1 to S7. [file 1471-2105-15-35-S2.pdf]
